# *Cryptosporidium* diagnosis in different groups of children and characterization of parasite species

**DOI:** 10.1590/0037-8682-0041-2022

**Published:** 2022-07-25

**Authors:** Flávia Thamiris Figueiredo Pacheco, Humberto Fonseca de Freitas, Renata Kelly Novais Rodrigues Silva, Silvia Souza de Carvalho, Adson Santos Martins, Joelma Figueiredo Menezes, Tereza Cristina Medrado Ribeiro, Ângela Peixoto de Mattos, Hugo da Costa-Ribeiro, Joice Neves Reis Pedreira, Neci Matos Soares, Márcia Cristina Aquino Teixeira

**Affiliations:** 1 Universidade Federal da Bahia, Programa de Pós-Graduação Stricto Sensu em Farmácia, Salvador, BA, Brasil.; 2 Universidade Federal da Bahia, Hospital Professor Edgard Santos, Salvador, BA, Brasil.; 3 Universidade Federal da Bahia, Departamento de Análises Clínicas e Toxicológicas, Salvador, BA, Brasil.

**Keywords:** Cryptosporidium, Diagnosis, Children, Genotyping

## Abstract

**Background:**

Microscopy and enzyme-linked immunosorbent assay (ELISA) are routinely used for *Cryptosporidium* diagnosis, without differentiating the parasite species.

**Methods:**

Children’s feces were analyzed by modiﬁed Ziehl-Neelsen (mZN) and ELISA for *Cryptosporidium* diagnosis and by polymerase chain reaction-restriction fragment length polymorphism for species identification.

**Results:**

*Cryptosporidium* frequency was 2.6%. The sensitivity and specificity of ELISA were 85.7% and 99.7%, respectively, with excellent concordance with mZN (kappa=0.854). Parasite species were characterized as *Cryptosporidium hominis* (78.3%), *Cryptosporidium felis* (17.4%), and *Cryptosporidium parvum* (4.3%).

**Conclusions:**

Coproantigen ELISA is as efficient as mZN for *Cryptosporidium* diagnosis. *Cryptosporidium* genotyping suggests anthroponotic and zoonotic transmission to children.

Laboratories testing for *Cryptosporidium* often use low-sensitive diagnostic methods that usually rely on the recognition of the *Cryptosporidium* oocysts in stool specimens stained by modified Ziehl-Neelsen (mZN). However, parasitological diagnosis has several limitations: it is time-consuming, requires skilled microscopists to identify the oocysts, and has low sensitivity when there are fewer oocysts in fecal samples^1^ ^,^ ^2^. Consequently, immunological methods have been developed for both clinical and environmental monitoring, and they seem to be more sensitive than the routine parasitological techniques^3^ ^,^ ^4^.

Although widely used for *Cryptosporidium* diagnosis, microscopy or coproantigen detection does not enable the differentiation of parasite species, which is an important technical limitation. The diagnosis and genetic characterization of the different species of *Cryptosporidium* are essential for the prevention, surveillance, and control of cryptosporidiosis^5^. Several *Cryptosporidium* species causing disease in humans have been described; however, *C. hominis* and *C. parvum* species are the predominant human infective species in most countries^6^. 

In Brazil, studies of *Cryptosporidium* genotyping are still scarce, and thus far, no such studies were conducted in Bahia, a state in the Brazilian Northeast region. Therefore, this study aimed to compare mZN staining with coproantigen detection for *Cryptosporidium* diagnosis in fecal samples of different pediatric groups and to characterize the *Cryptosporidium* species isolated in our laboratory.

Fecal samples were collected from 626 children from Salvador, Bahia, Brazil: 258 children from daycare centers, 171 with diarrhea, 88 with severe malnutrition, 79 with cancer, and 30 human immunodeficiency virus (HIV)-seropositive. Children admitted to the Hospital of Federal University of Bahia, Brazil, comprised the malnutrition and diarrhea pediatric groups. Children with cancer or HIV were outpatients of the same hospital, assisted by ambulatory or laboratory services. Children recruited from two daycare centers were apparently healthy. Fecal samples, except for the HIV group, were obtained as part of the *Giardia duodenalis* diagnosis study^7^ conducted at the Laboratory of Parasitology, Pharmacy College, Federal University of Bahia. For molecular characterization of *Cryptosporidium* species, we included nine positive extra-fecal samples, diagnosed during laboratory routine analysis: two from daycare children and two from adults with HIV, residents of Salvador, Bahia, and five from children from a rural area of a small city on the north coast of Bahia. The Ethics Committee of Nursing School, Federal University of Bahia, Brazil, approved the study (project approval no. 907.867).

Fecal samples were concentrated by centrifugal-sedimentation and stained by mZN for oocyst identification. A commercial enzyme-linked immunosorbent assay (ELISA; *Cryptosporidium* II, TECHLAB, Blacksburg, VA, USA) was used for the detection of *Cryptosporidium* antigens in stools. Thereafter, all *Cryptosporidium*-positive samples were subjected to DNA extraction for the molecular characterization of parasite species by polymerase chain reaction (PCR)-restriction fragment length polymorphism (RFLP) of *Cryptosporidium* oocyst wall protein (COWP) and the 18S ribosomal DNA (18S rDNA) targets.

Oocyst disruption and DNA purification were performed using QIAamp DNA Stool Mini Kit (Qiagen GmbH, Germany). Nested-PCR at COWP locus was performed as described by Pedraza-Díaz et al.^8^ For the primary PCR assay, a fragment of approximately 769 bp of the COWP gene was amplified with primers BCOWP-F and BCOWP-R (Invitrogen, Spain). A second PCR assay amplified the 553 bp DNA fragment. For the 18S rDNA nested-PCR, a primary 1,325 bp PCR amplicon was amplified using the primers 18S-F and 18S-R (Invitrogen, Spain). For the secondary PCR assay, the first-PCR product diluted 1:100 and primers 18SN-F and 18SN-R (Invitrogen, Spain) were used to amplify the 819-825 bp DNA product^9^. All the PCR products were subjected to electrophoresis (100 volts for 90 min) on ethidium bromide-stained 1% agarose gels and analyzed with an ultraviolet transilluminator (Loccus, Biotecnologia, Brazil). 

Secondary amplicons were digested using the restriction enzymes *Rsa*I (COWP) and *Ssp*I and *Vsp*I (18S) (Fermentas, Thermo Fisher Scientific), at 37°C for 16 h, for RFLP analysis. DNA fragments were subjected to 3% agarose gel electrophoresis (100 volts for 2 hours) and analyzed as described above. Samples whose RFLP analysis suggested *C. felis* infection or *C. hominis*/*C. felis* mixed infections had their 18S rDNA amplicons purified and sequenced in both directions by the Macrogen Inc. (Macrogen Inc., Seoul, Korea) sequencing service. *C. felis* nucleotides were aligned with reference sequences and deposited in the GenBank under accession codes MN174090 to MN174093.

For comparative evaluation of diagnostic techniques, the sensitivity and specificity of the ELISA were determined considering mZN as the gold standard. The agreement between methods was evaluated with Cohen’s Kappa coefficient, and discordant results were compared with the parasite DNA amplification by PCR assay. The differences in *Cryptosporidium* frequencies among pediatric groups were evaluated using chi-square test.

Gender distribution was homogeneous between pediatric groups, and regarding age range, 89.0% of children were under 5 years old (Table 1). The majority of families (69.5%) had a monthly income of less than or equal to a Brazilian minimum wage, and this variable reached 84.8% in the cancer patient group (Table 1), which highlights the socioeconomic vulnerability of this group. Socioeconomic variables could not be analyzed for the HIV group because these children were referred by the Child Support Court of Bahia and lived in an institution supported by a non-governmental organization (House of Support and Assistance for People with HIV/AIDS). Concerning the sanitary conditions of residences, more than 90% of the children’s families had access to potable water and sewage system, which is expected because most participants lived in urban areas with basic sanitation amenities. One exception was the cancer group, which had lower frequencies of piped water and bathroom with sink (*P*<0.01). Domestic animals were present in 31.9% of households in general, but when analyzing separately, the frequency was higher in children with cancer than in those with diarrhea and those from daycare (65.8%; *P*<0.01; Table 1).

An analysis of 626 stools by mZN and ELISA revealed the presence of *Cryptosporidium* oocysts in 16 samples, corresponding to an overall occurrence of 2.6% of parasites in the children examined (Table 1). The highest frequency of *Cryptosporidium* infection was observed in children with diarrhea (4.7%) and the lowest in asymptomatic daycare children (1.2%).

**TABLE 1:** Demographic, socioeconomic, and sanitary characteristics and frequency of *Cryptosporidium* infection in pediatric groups.

	Groups of children, N (%)					
	Diarrhea	Malnourished	Cancer	HIV-seropositive	Daycare	Total
	171 (27.3)	88 (14.1)	79 (12.6)	30 (4.8)	258 (41.2)	626 (100.0)
**Sex**						
Female	69 (40.4)	47 (53.4)	36 (45.6)	17 (56.7)	114 (44.2)	283 (45.2)
Male	102 (59.6)	41 (46.6)	43 (54.4)	13 (43.3)	144 (55.8)	343 (54.8)
**Age (years)**						
0-5	153 (89.5)^a,b^	81 (92.0)^a,b^	54 (68.4)^a,b,c^	11 (36.7)^a,b,c,d^	258 (100)^c,d^	557 (89.0)
5-10	18 (10.5)	7 (8.0)	25 (31.6)	19 (53.3)	0 (0.0)	69 (11.0)
**Residence area**						
Urban/periurban	156 (91.2)^a,b^	71 (80.7)^c^	47 (59.5)^a,c,d^	30 (100.0)^a,b,c,d^	258 (100.0)^a,d^	562 (89.8)
Rural	15 (8.8)	17 (19.3)	32 (40.5)	0 (0.0)	0 (0.0)	64 (10.2)
Highest education level of mother*						
None	2 (1.2)	2 (2.3)	6 (7.6)	----	0 (0.0)	10 (1.7)
Elementary school	112 (65.5)^a,c^	46 (52.2)^b,d^	29 (36.7)^a,b^	----	61 (23.6)^c,d^	248 (41.6)
High school	51 (29.8)^a,d^	38 (43.2)^b^	43 (54.4)^c,d^	----	191 (74.0)^a,b,c^	323 (54.2)
College	6 (3.5)	2 (2.3)	1 (1.3)	----	6 (2.3)	15 (2.5)
Monthly income (MW)*						
≤1 MW	110 (64.3)	68 (77.3)	67 (84.8)^a^	----	169 (65.5)^a^	414 (69.5)
>1≤2 MW	45 (26.3)	15 (17.0)	8 (10.1)	----	68 (26.4)	136 (22.8)
>2 MW	16 (9.4)	5 (5.7)	4 (5.1)	----	21 (8.1)	46 (7.7)
**Access to sanitary conditions/pets**						
Piped water	165 (96.5)^a^	80 (90.9)^b^	53 (67.1)^a,b,c,d^	30 (100.0)^c^	249 (96.5)^d^	577 (92.2)
Sewage system	146 (85.4)^a^	59 (67.1)^b^	38 (48.1)^a,c,d^	30 (100.0)^c^	234 (90.7)^d^	507 (81.0)
Bathroom with sink	150 (87.7)^a^	73 (83.0)^b^	54 (68.4)^a,b,c,d^	30 (100.0)^c^	253 (98.1)^d^	560 (89.5)
Contact with dogs and/or cats	58 (33.9)^a^	25 (28.4)^b^	52 (65.8)^ac^	0 (0.0)	65 (25.2)^c^	200 (31.9)
***Cryptosporidium* infection**						
	8 (4.7)^a^	2 (2.3)	2 (2.5)	1 (3.3)	3 (1.2)^a^	16 (2.6)

**MW:** minimum wage; **HIV:** human immunodeficiency virus. *The total number of children analyzed for the variables education level of mothers and family income was 596, as they were not evaluated for the HIV group. ^a,b,c,d^Equal letters indicate differences (*P*<0.01, χ^2^ test) in the frequency of the variables analyzed among groups.

*Cryptosporidium* was identified in 14 samples (2.2%) by both mZN staining and ELISA; two samples were diagnosed exclusively by microscopy and two by ELISA (Table 2). The sensitivity and specificity of ELISA were 85.7% and 99.7%, respectively, compared with those of mZN. The concordance between the diagnostics methods was considered excellent (Kappa = 0.854). Although there was a high agreement between mZN and ELISA for parasite detection in feces, both methods failed to diagnose two samples. To investigate these differences and exclude false-positive results, we compared the data with those of the parasite DNA amplification by PCR. For samples identified exclusively by ELISA (D8 and R3) or diagnosed only by microscopy using mZN (D1 and M2), at least one of the genes analyzed were amplified. On the contrary, the sample R4, classified as positive for *Cryptosporidium* by mZN and ELISA, showed a negative result on PCR (Table 3).

**TABLE 2:** Concordance between ELISA and mZN for *Cryptosporidium* diagnosis in feces.

	Modiﬁed Ziehl-Neelsen			Sensitivity*	Specificity*	Kappa
ELISA	Positive	Negative	Total	(95% CI)	(95% CI)	(95% CI)
Positive	12	2	14	85.7%	99.7%	0.854
Negative	2	610	612			
Total	14	612	626	(60.1-96.0%)	(98.8-99.9%)	(0.725-0.995)

**ELISA:** enzyme-linked immunosorbent assay; **CI:** confidence interval; *Sensitivity and specificity of ELISA were determined by considering mZN as the gold standard. Total frequency of *Cryptosporidium* by combining both methods was 2.6% (n=16).

**TABLE 3:** Origin of *Cryptosporidium* samples, diagnosis method, and molecular characterization of parasite species by analysis of COWP and 18S rDNA genes.

Origin of samples	Samples	ELISA	Modiﬁed Ziehl-Neelsen	COWP	***Cryptosporidium* species**	18S rDNA	***Cryptosporidium* species**
Children with diarrhea	**D1**	-	+	+	*C. hominis*	+	*C. hominis*
	**D2**	+	+	+	*C. hominis*	+	*C. hominis*
	**D3**	+	+	+	*C. hominis*	+	*C. hominis*
	**D4**	+	+	+	*C. hominis*	+	C. felis*
	**D5**	+	+	+	*C. hominis*	+	C. felis*
	**D6**	+	+	+	*C. hominis*	+	*C. hominis*
	**D7**	+	+	+	*C. hominis*	+	*C. hominis*
	**D8**	+	-	+	*C. hominis*	+	*C. hominis*
Malnourished children	**M1**	+	+	+	*C. hominis*	+	*C. hominis*
	**M2**	-	+	+	*C. hominis*	-	NA
Children with cancer	**C1**	+	+	+	*C. hominis*	+	*C. hominis*
	**C2**	+	+	+	*C. hominis*	+	*C. hominis*
HIV-seropositive children	**CH1**	+	-	-	NA	-	NA
Daycare children	**DC1**	+	+	-	NA	+	C. felis*
	**DC2**	+	+	+	*C. hominis*	+	*C. hominis*
	**DC3**	+	+	+	*C. hominis*	+	*C. hominis*
	DC4	+	+	+	*C. hominis*	+	*C. hominis*
	**DC5**	+	+	+	*C. hominis*	+	*C. hominis*
Children from rural area	**R1**	+	+	+	*C. hominis*	+	*C. hominis*
	**R2**	+	+	+	*C. hominis*	+	*C. hominis*
	**R3**	+	-	+	*C. hominis*	+	*C. hominis*
	**R4**	+	+	-	NA	-	NA
	**R5**	+	+	+	*C. parvum*	+	*C. parvum*
HIV-seropositive adults	**AH1**	+	+	+	*C. hominis*	+	*C. hominis*
	**AH2**	+	+	-	NA	+	C. felis*

**ELISA:** enzyme-linked immunosorbent assay; **COWP:** *Cryptosporidium* oocyst wall protein; **18S rDNA:** 18S ribosomal DNA; **+:** positive; **-:** negative; **NA:** no DNA amplification; **HIV:** human immunodeficiency vírus. * *C. felis* isolates were further confirmed by 18S rDNA sequencing.

Combining the results of amplification of both genes analyzed, the PCR had an efficiency of 92% (n= 23), although the 18S locus was more effective for genotyping. Of the 21 samples wherein the COWP gene was amplified, 20 (95.2%) exhibited a pattern of *C. hominis* and one isolate (1.8%) had a pattern of *C. parvum*. From the 22 isolates that amplified the 18S rDNA gene, 17 (77.3%) exhibited a *C. hominis* pattern, 1 (4.5%) had a *C. parvum* pattern, and 4 (18.2%) had a *C. felis* pattern (Table 3). From the 20 samples with amplification at both loci, 18 (90.0%) showed agreement on *Cryptosporidium* species characterization by RFLP, but two samples (D4 and D5) identified as *C. hominis* by COWP gene analysis showed a *C. felis* profile at 18S locus (Table 3). The four samples suggestive of *C. felis*, which were further subjected to 18S rDNA sequencing, confirmed all cases. Therefore, considering the 23 isolates characterized, the final distribution of *Cryptosporidium* species was 78.3% of *C. hominis* (n=18), 17.4% of *C. felis* (n=4), and 4.3% of *C. parvum* (n=1). *C. parvum* was observed only in children from a rural area and *C. felis* was found in both children with diarrhea and those from daycare and in one adult patient with HIV (Table 3).

Many studies suggest that ELISA has better performance for *Cryptosporidium* diagnosis than parasitological methods such as mZN staining^3^ ^,^ ^10^. The efficiency of diagnostic methods is mainly influenced by the parasite load in feces. Because of intense shedding of oocysts in diarrhea patients, microscopy may produce similar results as those of antigen detection. Conversely, in asymptomatic patients with low parasite shedding, parasitological methods can yield false-negative results, depending on the fecal concentration method used and the microscopist expertise^1^. In this study, despite the origin of fecal samples from different pediatric groups, with or without diarrhea, ELISA showed high sensitivity and specificity, 85.7% and 99.7%, respectively, but detected the same number of *Cryptosporidium*-positive feces and missed two samples detected only by microscopy, later confirmed by PCR assay. This failure may be explained by the non-accessibility or recognition of the antigen by the monoclonal antibody, or even degradation of antigenic epitopes^3^. Therefore, according to our results, ELISA did not show a significant improvement in parasite diagnosis when compared with mZN. Noteworthy, despite the high sensitivity of PCR, two *Cryptosporidium-*positive feces, for at least one of the diagnostic methods, did not amplify any of the genes analyzed. The lack of amplification may be accounted for the presence of polymerase inhibitors in the feces and degradation or mutations of *Cryptosporidium* DNA in these samples^11^.

Owing to the ability of *Cryptosporidium* to infect various animals, humans may acquire infections through anthroponotic or zoonotic routes^6^ ^,^ ^12^. In this study, *C. hominis* (87%) was the most frequently identified species, indicating that the anthroponotic transmission of *Cryptosporidium* is more prevalent in urban environments, which agree with findings from studies conducted in other Brazilian states such as in Rio de Janeiro (73.7%) and São Paulo (63%)^13^ ^,^ ^14^. Conversely, in children from an urban favela in Fortaleza, another metropolitan city of the Brazilian Northeast, there was no significant difference between frequencies of *C. hominis* and *C. parvum* ^15^, which may be related to precarious sanitary conditions of favelas, with transmission of oocysts from humans and animals and spread across in the environment. In this study, one isolate was identified as *C. parvum* in a child from a rural area of a small coastal town, where close contact with animals is very common. Moreover, we found four isolates of *C. felis*, a species with the domestic cat as the main host, that eventually infects humans, especially patients with AIDS^14^, and it is rarely reported in immunocompetent children. From the four individuals infected with *C. felis*, one was an adult with HIV, two were children hospitalized for diarrhea, and another was an asymptomatic daycare child, suggesting regular transmission of this parasite species in different patient groups in Salvador.

In conclusion, this study showed that despite the high sensitivity and specificity of coproantigen ELISA, it should not be used as the sole method for *Cryptosporidium* diagnosis, as similar results can be obtained using mZN, considering its low cost in resource-poor settings. In reference or research laboratories of developing countries, the PCR-RFLP can be useful to check disagreement between microscopy and ELISA and to determine *Cryptosporidium* species. Few studies in Brazil have characterized *Cryptosporidium* isolated from children, lacking epidemiological data about the susceptibility of children according to the parasite species. In our study, the most frequent species was *C. hominis*, suggesting that the anthroponotic transmission route for *Cryptosporidium* is more common in children of Salvador, Bahia. Nevertheless, the identification of *C. felis* cases highlights the role of cats in the zoonotic transmission of this coccidian. The results also indicate that *C. felis* seems to be the second species more involved in *Cryptosporidium* transmission in Salvador and that it does not require HIV coinfection. 
